# Regenerative Applications Using Tooth Derived Stem Cells in Other Than Tooth Regeneration: A Literature Review

**DOI:** 10.1155/2016/9305986

**Published:** 2015-12-20

**Authors:** Yun-Jong Park, Seunghee Cha, Young-Seok Park

**Affiliations:** ^1^Department of Oral and Maxillofacial Diagnostic Sciences, College of Dentistry, University of Florida, Gainesville, FL 32610, USA; ^2^Department of Oral Anatomy, Dental Research Institute and School of Dentistry, Seoul National University, Seoul 03080, Republic of Korea

## Abstract

Tooth derived stem cells or dental stem cells are categorized according to the location from which they are isolated and represent a promising source of cells for regenerative medicine. Originally, as one kind of mesenchymal stem cells, they are considered an alternative of bone marrow stromal cells. They share many commonalties but maintain differences. Considering their original function in development and the homeostasis of tooth structures, many applications of these cells in dentistry have aimed at tooth structure regeneration; however, the application in other than tooth structures has been attempted extensively. The availability from discarded or removed teeth can be an innate benefit as a source of autologous cells. Their origin from the neural crest results in exploitation of neurological and numerous other applications. This review briefly highlights current and future perspectives of the regenerative applications of tooth derived stem cells in areas beyond tooth regeneration.

## 1. Introduction

Stem cells are undifferentiated cells defined by two distinct characteristics, namely, their ability to continuously self-renew and to differentiate into multiple mature cell types [1]. Stem cells are significant because one stem cell has the potential to serve as an inexhaustible proliferative source for replacement therapy, comprising one component of the tissue engineering triad [2]. Stem cells are considered by both researchers and members of the public alike to represent one of the best hopes for regenerating irreversibly damaged tissue that cannot be repaired by contemporary medical approaches [3]. Indeed, regenerative medicine and emerging biotechnologies are revolutionizing the practice of medicine and the ensuing advancements are helping to propel stem cells for tissue regeneration into a clinical reality [4].

Stem cells can be categorized as embryogenic stem cells (ESCs) or adult stem cells according to the stage at which they are isolated. Embryogenic stem cells are totipotent, being derived from the inner cell mass of blastocysts during gastrulation [5]. Although they have the greatest biological potential, ethical issue on the use of ESCs has precluded their widespread study, especially in humans. Naturally then, the focus has moved towards adult stem cells, which are derived from postnatal fully developed tissue [6] and they are thought to renew cell populations, maintain tissue homeostasis, and participate in tissue repair following injury [3]. Compared with ESCs, adult derived stem cells have several limitations with respect to lifespan and differentiation potential [7].

Recently, induced pluripotent stem cells (iPSCs) were developed, which are reprogrammed somatic cells having pluripotency-like embryonic stem cells [8]. This might provide an alternative pathway that might eliminate the ethical issues regarding use of tissue from human embryos and allow us to overcome problems of rejection after nonautologous cell implantation. Therefore, they were expected to become the important tool in the advancement of personalized medicine [9]. Basically, iPSCs have been generated by reprograming cells via incorporation of several genes and they have similarities to human ESCs in their morphologies, gene expression,* in vitro* differentiation potential, and teratoma formation. To date, many researchers have reported that iPSCs can differentiate into different cell types, such as neuron [10], pancreas [11], cardiac myocytes [12], and renal lineage cell [13] under appropriate condition. As a tooth related structure, differentiation of dental epithelial-like cells was firstly induced from the mouse ES cell using culture methods with ameloblasts serum-free medium [14]. Several reports have subsequently proposed and demonstrated that iPSC can differentiate into tooth related cells including dental mesenchymal cells for regeneration purpose [15–19]. In this respect, the iPSCs are expected to serve as a new scientific stream of material for tissue regeneration [20].

Notably, iPSC technology itself requires the most suitable cell sources. Until now, various human cell sources have been demonstrated to be reprogrammed to iPSCs, including dermal fibroblasts, skin keratinocytes, amniotic fluid-derived cells, embryonic stem cell-derived fibroblasts (ESFs), CD34 blood cells, and mesenchymal stem cells (MSCs) [21]. In addition to these, dental pulp cells have recently been shown as a new rich source for iPSC technology [22]. They are known to have several advantages over preexisting sources in terms of reprogramming efficiency, multipotency, technical feasibility, and accessibility [23, 24]. Therefore, iPSCs made from dental cells can provide more powerful tools for regenerative application in other than tooth structures before long hopefully, which could be described in future reviews. However, scientists should answer the question whether iPSCs are truly equal to ES cells. Furthermore, the tendency for tumor generation after iPSC transplantation is one of the greatest concerns as of now. Our current knowledge about iPSC variability and its manipulating technology must be greatly improved before iPSC became standard regenerative tools.

Mesenchymal stem cells (MSCs) are one of the most widely studied types of adult stem cells. MSCs were first identified by Friedenstein et al. as self-renewing fibroblast-like cells in bone marrow [25] and were initially referred to as bone marrow stem cells (BMSCs) and bone marrow mesenchymal stem cells (BMMSCs). Owing to the various techniques for isolating, expanding, and characterizing MSCs, a minimal set of criteria for identifying these cells was suggested by the Mesenchymal and Tissue Stem Cell Committee of the International Society for Cellular Therapy [26]. Specifically, MSCs (1) must be plastic-adherent under standard culture conditions, (2) express CD105, CD73, and CD90, and (3) lack expression of CD45, CD34, CD14 or CD11b, CD79*α* or CD19, and HLA-DR surface molecules. Based on these criteria, MSCs are able to differentiate into osteogenic, chondrogenic, and adipogenic lineages.

To date, a tremendous amount of knowledge regarding stem cell biology has been gained from studying BMSCs. At the same time, significant efforts have been made towards identifying different sources of MSCs other than bone marrow, since there are a number of inconveniences associated with acquiring BMSCs, including pain and morbidity. Furthermore, the number of viable cells obtained from harvesting bone marrow is often insufficient for downstream purposes [27].

In this regard, dental stem cells may represent a good alternative to BMSCs due to the ease with which they can be obtained and lack of morbidity at the donor site. Tooth derived stem cells can be classified according to the part of the tooth from which they are isolated, namely, (1) dental pulp stem cells (DPSCs) [28], (2) stem cells from human exfoliated deciduous teeth (SHEDs) [29], (3) periodontal ligament stem cells (PDLSCs) [30], (4) stem cells from apical papilla (SCAPs) [31], and (5) dental follicle progenitor cells (DFPCs) [32]. Although they are all derived from tooth related structures, the specific properties of these different dental stem cell populations such as expression markers and differentiation potencies are slightly different according to the location from which they are isolated.

**Figure 1:**
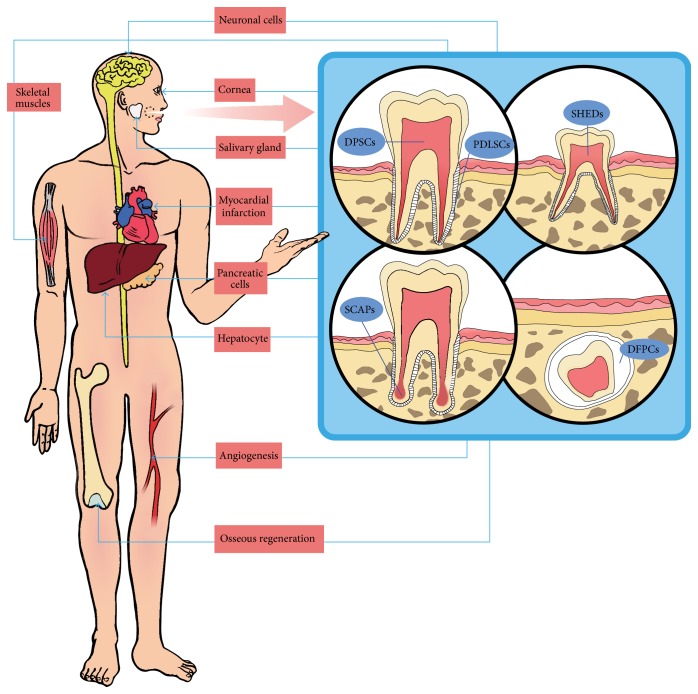
Regeneration of various tissues or organs from tooth derived stem cells. Stem cells from tooth related structures can be used to regenerate not only dentin-pulp complex but also various tissues or organs of body, such as bone, vascular system, nerve, liver, pancreas, salivary glands, skeletal muscle, and cornea. DPSCs (dental pulp stem cells), PDLSCs (periodontal ligament stem cells), SHEDs (stem cells from human exfoliated deciduous teeth), SCAPs (stem cells from apical papilla), and DFPCs (dental follicle progenitor cells).

The majority of research employing dental stem cells has been directed towards regeneration of damaged tooth related structures, either partially or in its entirety [33]. Indeed, the findings of a substantial number of studies have garnered increasing interest in dental pulp regeneration [34, 35], that is, regenerative endodontic procedures that may be able to change the fundamental paradigm of endodontics [36]. Likewise, regeneration of the periodontal complex has also been investigated [37]. In addition to these applications, tooth derived stem cells have been studied as a potential source for tissue regeneration purposes beyond tooth related structures, similar to MSCs. One well-known example is osseous regeneration, including craniofacial or alveolar bone, in which the field of dentistry has great interest. In support of this possibility, several attempts have been made to generate various human tissues from tooth derived stem cells, with some studies yielding impressive outcomes (Figure 1).

This review article aims to provide a summarized overview of the medical applications of tooth derived stem cells for tissues other than tooth related structures and discuss future perspectives of tooth derived stem cells in the context of regenerating tissues from diverse origins.

## 2. Brief Reviews on the Types of Tooth Derived Stem Cells

Various types of tooth derived stem cells have been utilized in the field of regeneration medicine. As it was briefly mentioned earlier, the advantages of tooth derived stem cells in comparison with BMSCs include but are not limited to accessibility with little or no morbidity of donor site, high proliferation rate, and multipotency [38].

### 2.1. Dental Pulp Stem Cells (DPSCs)

DPSCs are the first tooth derived stem cells in 2000 and are mesenchymal type of cells inside dental pulp [28]. DPSCs are known to differentiate into several kinds of cells and tissues, such as osteoblast, smooth muscle cells, adipocyte-like cells, neuron, dentin, and dentin-pulp-like complex [3]. They were also shown to have chondrogenic potentials* in vitro*. Their multipotency, proliferation rate, availability, and cell number have been demonstrated to be greater than those of BMSCs. Overall, DPSCs are more suitable than BMSCs for mineralized tissue regeneration [39].

### 2.2. Stem Cells from Human Exfoliated Deciduous Teeth (SHEDs)

SHEDs are progenitor cells isolated from the pulp remnant of exfoliated deciduous teeth. Interestingly, they showed more proliferation rate and higher capability for differentiation than BMSCs and even DPSCs in a number of studies [29, 40]. Osteoblast, odontoblast, adipocyte, and neural cells have been reported to be differentiated from SHEDs [3].

### 2.3. Periodontal Ligament Stem Cells (PDLSCs)

Although periodontal ligaments are known to be of neural crest cell origin, PDLSCs exhibit stem cell characteristics similar to MSCs [41]. In addition, PDLSCs residing in the perivascular wall have common properties in cell morphology, phenotype, and differentiation potentials [42]. Immunomodulatory ability is another feature resembling BMSCs [43]. PDLSCs are able to differentiate into osteoblast, cementoblasts, adipocytes, and chondrocyte and they were reported to form periodontal ligament and cementum-like tissue* in vivo* [37].

### 2.4. Stem Cells from Apical Papilla (SCAPs)

SCAPs are cells isolated from the root apex of developing tooth, which is thought to be associated with root formation [31]. They present the characteristics of MSCs and can differentiate into osteoblast, adipocyte, chondrocyte, and neuron under appropriate conditions [44].

### 2.5. Dental Follicle Progenitor Cells (DFPCs)

DFPCs are stem cells extracted from dental follicle surrounding tooth germ in early tooth formation stages [32]. The dental follicle is an ectomesenchymal cell condensation and harbors heterogeneous population of cells comprising periodontium. They are also known to be differentiated into osteoblast, adipocyte, chondrocyte, and neuronal cells [3].

Because DPSCs are the most studied, we will focus mainly on usage of DPSCs herein and report on SHEDs and PDLSCs will also be discussed. However, it does not mean that any kind of tooth derived stem cell is superior to or more promising than others in regeneration medicine. We need substantial amount of further study to talk about that.

## 3. Osseous Regeneration

Having an ectomesenchymal origin, DPSCs contain bone-specific markers and exhibit an osteogenic differentiation profile [45–47]. Upon differentiating into preosteoblasts, DPSCs deposit an extracellular matrix that eventually forms mineralized woven bone [48]. Graziano et al. showed that CD34+ DPSCs transplanted in the subcutaneous tissue of rats form a substantial number of bone nodules [49]. Due to the superior efficiency in producing bone chips compared with BMSCs, DPSCs are considered one of the best candidates for bone regeneration [50]. Consistently, the reconstruction of large-scale cranial bone defect was reported in nonimmunocompromised rats [51]. In a clinical study, biocomplexes prepared from DPSCs and collagen sponges were used in human mandible repair and exhibited impressive results [52]. In conjunction with other biomaterial platforms, DPSCs have been shown to have osteogenic differentiation capacity [53–56]. The topography of scaffolds was also reported to play a crucial role in clinical regeneration [57].

With respect to alveolar bone defects, Liu et al. showed that DPSCs expressing bone morphogenic protein 2 (BMP-2) undergo earlier mineralization and generate a greater amount of bone in a rabbit model [58]. Prior to this observation, evidence regarding the effect of BMP-2 on the osteoinducibility of DPSCs was presented in several studies [59–61]. Platelet rich plasma has also been evaluated in the same context [62, 63].

The role of DPSCs in bone regeneration around dental implants was recently investigated [63], and a similar study performed with BMSCs and periosteal cells showed that DPSCs exhibit the highest osteogenic potential as a source for tissue-engineered bone around titanium implants [64]. Interestingly, a recent report suggested that immobilization of DPSCs in alginate hydrogels results in enhanced osteogenic potential compared with control cells cultured in conventional stem cell media [65].

Osseous regeneration using PDLSCs has been investigated in a number of studies. Chadipiralla et al. compared the* in vitro* proliferation and calcium deposition of PDLSCs with SHEDs under retinoic acid treatment with insulin and found that PDLSCs exhibit superior properties [66]. Regarding alveolar bone regeneration, PDLSCs demonstrated promising results in swine and canine models, although these results are not confined to osseous tissue alone and include periodontal tissues [67, 68]. The effect of adding different scaffolds such as *β*-TCP, bovine bone mineral, and hydroxyapatite chitosan has also been investigated. In addition, increased new bone formation and reosseointegration around peri-implantitis defects using* ex vivo* BMP-2 gene delivery in PDLSCs were reported [69]. Collectively, these studies suggest that PDLSCs represent a promising tool for bone regeneration [70–72].

Bone and dentin are both calcified tissues made from mesenchymal cells of the same embryogenic origin. Thus, they share many biochemical and molecular characteristics. Extracellular matrix of both tissues is highly mineralized with deposition of hydroxyapatite crystals. However, there exist differences between them [73]. While bones undergo constant remodelling, dentin is not remodelled but continuously deposited [74]. Tooth derived stem cells can differentiate and then produce either dentin or bone-like tissue according to the environment such as scaffolds, growth factors, mechanical loading, and the combinations of them. Usually and unintentionally, the bone-like tissue formation occurs simultaneously with the dentin-like tissue formation in the tooth derived stem cell transplanted samples. Basically, they form mineralized tissues with odontoblastic differentiation only under special environments, such as in the existence of intact dentin [75]. Considering the tooth development process in which epithelial mesenchymal interaction is cardinal features for both ameloblast and odontoblast differentiation [76], the odontoblast differentiation is presumably difficult without signals from ameloblasts. Interestingly, other mesenchymal stem cells like BMSCs and adipose derived stem cells (ADSCs) can also produce dentin-like structures, but the results are not the same. These stem cells seem to differ in plasticity and possess a “positional memory” [77].

## 4. Neural Regeneration

Despite limited amount of adult neural stem cells damage caused by harvesting cells, neural regeneration is considered imperative, since many nerve degeneration related diseases have no effective remedies and the sequelae are usually catastrophic. Thus, there have been tremendous attempts to identify appropriate stem cells with neural potentials. DPSCs exhibit markers of multipotency, several of which are associated with spontaneous neural differentiation [78]. Thus, DPSCs stand out as strong candidates as a source of neural stem cells, which are in contrast to BMSCs, which have a low differentiation efficiency. Considering the origin of dental pulp tissue, it may not be surprising that DPSCs exhibit intrinsic neuroglial characteristics and are capable of differentiating into both neural and vascular endothelial cells [79]. Consistent with this observation, a significant subpopulation of DPSCs was recently demonstrated to have glial origins [80].

DPSCs express several neural markers upon appropriate stimulation in neural differentiation media, and a number of studies have reported successful* in vitro* formation of neurospheres and effective neuronal induction [79, 81–84]. Importantly, these findings have been confirmed* in vivo* [85, 86], along with reports of cell-mediated neuroplasticity and endogenous neural stem cells recruitment by DPSCs [87, 88]. Recently, regulation of the differentiation of DPSCs into different neuronal phenotypes was investigated at the molecular level [89]. In addition, differentiated murine DPSCs were shown to form immature neuronal-like networks [90].

Studies have attempted neural regeneration with dental pulp-derived cells (DPCs) far earlier than those with DPSCs. Nosrat et al. [91] demonstrated that DPC grafting promotes survival of damaged motor neurons in a rat model of spinal cord injury. In a subsequent report, several neurotrophic factors like nerve growth factor (NGF), brain-derived neurotrophic factor (BDNF), and glial cell line-derived neurotrophic factor (GNDF) were shown to be secreted from DPCs, which in turn facilitated survival of sensory and dopaminergic neurons. This neurotrophic effect was confirmed later using* in vitro* models of Alzheimer's and Parkinson's disease [92]. DPCs have also been used in regeneration of injured peripheral nerves [93, 94]. Importantly, although these studies were performed using DPCs, DPSCs are expected to exhibit many of the same properties [35].

The secretion of neurotrophic factors from transplanted DPSCs has been suggested to provoke a kind of chain reaction that induces neighboring cells to differentiate and secrete other neurotrophic factors important to the repair of sites of injury [95–97]. Indeed, the neuroprotective roles of DPSCs have been investigated in several studies [98, 99]. In addition, DPSCs directly inhibit the activity of several axon growth inhibitors and prevent apoptosis of neurons, astrocytes, and oligodendrocytes. As another example of CNS regeneration, several studies have proposed stem cell therapy using DPSCs as a cure for stroke in a rodent model [100–103]. A recent review of spinal cord injury applications of DPSCs or SHEDs indicated that the high levels of proinflammatory mediators around transplanted cells may affect their fate towards an oligodendrocyte-specific differentiation cascade [104]. On the other hand, SHEDs were also reported to differentiate into dopaminergic neuron-like cells [40].

## 5. Treatment of Myocardial Infarction

Despite recent advances in prevention and treatment, myocardial infarction (MI) remains one of the cardinal causes of mortality worldwide. Several studies have investigated the possibility of using DPSCs for the treatment of MI. For example, Gandia et al. used human dental pulp stem cells (hDPSCs) in a rat myocardial infarction model in 2008, reporting an increase in the number of vessels and decrease in the size of infarct, concluding that hDPSCs secrete multiple proangiogenic apoptotic factors including VEGF [105]. In 2009, the overall capability of stem cells to differentiate into cells with a cardiac phenotype was evaluated including BMSCs, adipose tissue stem cells, and DPSCs [106].

## 6. Another Ischemic Disease (Angiogenesis)

Angiogenesis is defined as the formation of new blood vessel from preexisting blood vessels. If blood supply were not to be established properly and rapidly, there are insufficient oxygen and nutrient transportation and then necrosis of the implanted tissue will follow [107]. Vessel development in body has very complex and dynamic processes that are degradation of the basement membrane and the extracellular matrix (ECM), endothelial cell proliferation and migration, tube formation, and maturation into functional blood vessels [108, 109]. All these processes have been tightly modulated by an intricate balance of signals including growth factor and their receptor, several enzymes, matrix metalloproteinases (MMPs), cytokines, inhibitors, transcription factors, and adhesion molecules [109, 110]. To date, DPSCs have several advantages for clinical application compared with other types of adult stem cells because they can be easily obtained from extracted teeth. Moreover, DPSCs retain their multilineage differentiation capacity after cryopreservation [111]. In addition, recent studies indicate that DPSCs have a higher population rate with neural and epithelial stem cell properties than BMSCs [79].

Recently, a highly vasculogenic subfraction of DPSCs can be isolated from porcine dental pulp, which is similar to endothelial progenitor cells of dental pulp [112, 113]. Importantly, successful engraftment of these cells was observed upon transplanting this isolated cell population into the ischemic hind limb of mice. A number of studies have also discussed the endothelial differentiation of DPSCs [114–119]. Likewise, SHEDs have also been demonstrated to be able to differentiate into epithelial cells [120]. Concerning paracrine induction of angiogenesis, DPSCs were previously demonstrated to express a number of angiogenic factors such as platelet derived growth factor (PDGF), vascular endothelial growth factor (VEGF), and fibroblast growth factor (FGF) and were able to induce the tube formation of umbilical vein endothelial cells* in vitro* [121, 122]. Bronckaers et. al. showed that DPSCs produce high amount of angiogenic factors such as VEGF and MCP-1, and these also can stimulate endothelial cell migration* in vitro* by stimulating the PI3K/AKT and MEK/ERK pathway of endothelial cells. Moreover, in the* in vivo* CAM model DPSCs significantly induced the formation of blood vessels [123]. Therefore, DPSCs may represent an attractive stem cell source for tissue engineering and could be a treatment for inadequate angiogenesis such as chronic wounds, stroke, and myocardial infarction.

## 7. Hepatocyte Differentiation

Hepatocytes are the main cellular component of the liver, comprising 70–80% of the total liver mass. Ishkitiev et al. were the first researchers who showed the differentiation potential of DPSCs into hepatocyte-like cells [124]. The hepatic differentiation of cryopreserved hDPSCs was reported, as well as their functional glycogen storage and urea production abilities [125]. More recently, several studies have provided strong evidence to support a role for DPSCs in the treatment of irreversible liver disease, providing hope for a future cure [126–129].

## 8. Corneal Reproduction

Reconstruction of the cornea using DPSCs has been explored in recent years. In one animal study, a tissue-engineered sheet of DPSC was transplanted on the corneal bed and deepithelialized human amniotic membrane covering was done. Healthy uniform corneal epithelium was formed after three months of healing [130]. DPSCs isolated from third molars were also reported to be capable of differentiating into keratocytes, which are cornea stroma cells [131].

In addition to cornea reconstruction, human PDLSCs may also be directed towards retinal progenitors having competence for photoreceptor differentiation [132]. Oral mucosal epithelial cells have also been investigated in the context of ocular surface reconstruction [133]. Lastly, although categorized as neural regeneration in a strict sense, intravitreally transplanted DPSCs have been reported to promote regeneration of retinal ganglion axon following optic nerve crash injury [134].

## 9. Treatment of Diabetes

Diabetes is more and more increasing these days and becomes one of the most common chronic endocrinal diseases associated with pancreatic islet cell dysfunction. Not only type 1 but also type 2 diabetes may be successfully managed by transplantation of pancreatic islet cells. The differentiation potential of DPSCs into islet-like cell aggregates (ICAs) has been explored, with the results suggesting that* in vitro* cultured ICAs can release insulin and C-peptide in a glucose-dependent manner [135]. Carnevale et al. also reported that human amniotic fluid stem cells and hDPSCs differentiate into insulin-producing cells, suggesting their potential as a nonpancreatic, low-invasive source of cells for islet regeneration [136]. Furthermore, the physiological relevance of this technology was demonstrated with the generation of islet-like cell clusters derived from both DPSCs and SHEDs [137]. The differentiation potentials of DPSC and SHEDs into all functional endocrine and exocrine subsets of pancreatic cells were confirmed in a separate study [138], and diabetic sequelae were shown to be alleviated upon transplantation of DPSCs in a murine model [139]. Finally, the transdifferentiation potential of human PDLSCs cultured in Matrigel into pancreatic islet cells has been demonstrated [140].

## 10. Miscellaneous

Differentiation of DPSCs into skeletal muscle has been reported for the treatment of muscular dystrophy [141–143]. In addition, several studies have attempted to differentiate DPSCs into salivary gland cells [144].

Overall, studies indicate that DPSCs are more preferable than BM-MSCs for mineralized tissue regeneration [39, 145, 146]. However, differentiation of DPSCs into skeletal muscle has been reported for the treatment of muscular dystrophy [141–143] along with differentiation of DPSCs into salivary gland endothelial cells [143]. More specifically, a study by Kerkis et al. [141] utilized the golden retriever muscular dystrophy dogs for therapeutic trials of human immature DPSCs to target human Duchenne muscular dystrophy. Although statistical analyses could not be performed due to a small number of littermates for the study, the results indicated that DPSCs were significantly engrafted in GRMD dog muscles. Notably, the study also revealed that early transplantation of human immature DPSCs allowed no use of immune suppression and that effective multiple systemic deliveries of the cells increased effectiveness of engraftment. Nonetheless, authors acknowledged limitations of study based on their observations indicating modest human dystrophin expression and restricted expression only to several muscle fibers. Another study [142] regarding muscle regeneration indicated that DNA demethylation induced by 5-Aza treatment might trigger the skeletal muscle differentiation in mouse DPSCs.

As for the recovery of salivary glandular function in mice with irradiation, a study [144] reported that DPSCs isolated from GFP-expressing mice were differentiated into dental pulp endothelial cells (DPECs). DPECs showed typical endothelial morphology* in vitro* and* in vivo* when subcutaneously injected into nude mice. More interestingly, the mean saliva flow rate in mice treated with DPECs at 4 and 14 days after a single dose of 15 Gy irradiation was significantly higher than that observed in mice treated with PBS when measured 8 weeks after radiation. This study raises an interesting question by which molecular mechanisms these implanted DPECs served protective roles in secretory functions of irradiated mice, which was inconclusive in the study. Potential roles of secretory factors from DPECs or functional blood vessel formation by DPEC that promotes healing of radiation damage in the irradiated salivary glands can only be speculated as potential mechanisms at present time.

## 11. Future Perspectives

Despite the changing trends along the available technological advances, regenerative medicine can be defined as it replaces or regenerates human cells, tissue, or organs, to restore or establish normal functions [147]. Since there are a number of irreversible medical conditions that the current approaches cannot reverse, this field is more and more expanding with increasing interest [148]. Naturally, it encompasses wide variety of state-of-the-art medical principles including tissue engineering. The purpose of tissue engineering is to create the optimal condition to regenerate damaged tissues [149] and its basic triads of component are scaffolds [150], signaling molecules [151], and cells [152]. The cells, more specifically human cells, should be the central focus of regenerative medicine [147].

For cell-based regenerative therapy, the source of cells is a very important issue [153]. Harvesting procedures, accompanying morbidity, amount of cells, and its efficacy are all among the considerations [154]. In this regard, tooth derived stem cells have innate advantages. They were considered as an alternative of BMSCs but the different characteristics from difference in origin make them unique in the utilization as a cell source for regenerative medicine.

Although not a few studies have reported promising and sometimes impressive results of regenerations using tooth derived stem cells, there are also not a few hurdles to overcome for clinical applications. Interestingly and ironically as well, even the regeneration of origin structure, tooth, has not been accomplished in spite of ardent attempts [36]. Only the tooth-like structures are successfully formed; however they still cannot replace actual teeth and function like actual teeth in every aspect. Above all, the most appropriate cell type for regeneration of specific tissue has not been concluded. Furthermore, heterogeneous subpopulations exist even in the same cell type under the current identification criteria [155]. Clear identification, isolation, and purification are essential with more thorough understanding in their regulation mechanisms [156].

In addition, several limitations should be considered. Most of the regenerative studies have been done in animal models and evidences in human clinical research are still scanty. Like other stem cells, tooth derived stem cells are not free of concerns about tumorigenicity [157]. Finally, they are less potent than ESC in many aspects. Recently, reprogramming hDPSCs into iPS cells was introduced and this might solve at least partly the problem of potency [22, 158]. Tamaoki et al. suggested the suitability of DPCs as a bank source of iPS cells [159]. The attempt to use only nononcogenic factors to reprogram DPCs has been made [119].

## 12. Concluding Remarks

Tooth derived stem cells represent a viable source of adult stem cells for regenerative medicine. The unique origin of these cells can contribute to the regeneration of various tissue types, although their characteristics are slightly different according to the isolation location. DPSCs are the most studied cell type among them and regeneration of tooth structures is main topic. However, the tooth derived stem cells have shown their possibility as a cell source for nontooth structures, such as bone, nerve, muscle, liver, and pancreas. Their application will expand along the technological advances although our current information and experience are not enough. Subsequent researches are expected to be done with their fascinating benefit as a readily available cell source in the future.
